# Evaluation of the Agronomic Performance of Atrazine-Tolerant Transgenic *japonica* Rice Parental Lines for Utilization in Hybrid Seed Production

**DOI:** 10.1371/journal.pone.0108569

**Published:** 2014-10-02

**Authors:** Luhua Zhang, Haiwei Chen, Yanlan Li, Yanan Li, Shengjun Wang, Jinping Su, Xuejun Liu, Defu Chen, Xiwen Chen

**Affiliations:** 1 Laboratory of Molecular Genetics, College of Life Sciences, Nankai University, Tianjin, China; 2 Tianjin Crop Research Institute, Tianjin, China; Institute of Genetics and Developmental Biology, Chinese Academy of Sciences, China

## Abstract

Currently, the purity of hybrid seed is a crucial limiting factor when developing hybrid *japonica* rice (*Oryza sativa* L.). To chemically control hybrid seed purity, we transferred an improved atrazine chlorohydrolase gene (*atzA*) from *Pseudomonas* ADP into hybrid *japonica* parental lines (two maintainers, one restorer), and Nipponbare, by using *Agrobacterium*-mediated transformation. We subsequently selected several transgenic lines from each genotype by using PCR, RT-PCR, and germination analysis. In the presence of the investigated atrazine concentrations, particularly 150 µM atrazine, almost all of the transgenic lines produced significantly larger seedlings, with similar or higher germination percentages, than did the respective controls. Although the seedlings of transgenic lines were taller and gained more root biomass compared to the respective control plants, their growth was nevertheless inhibited by atrazine treatment compared to that without treatment. When grown in soil containing 2 mg/kg or 5 mg/kg atrazine, the transgenic lines were taller, and had higher total chlorophyll contents than did the respective controls; moreover, three of the strongest transgenic lines completely recovered after 45 days of growth. After treatment with 2 mg/kg or 5 mg/kg of atrazine, the atrazine residue remaining in the soil was 2.9–7.0% or 0.8–8.7% respectively, for transgenic lines, and 44.0–59.2% or 28.1–30.8%, respectively, for control plants. Spraying plants at the vegetative growth stage with 0.15% atrazine effectively killed control plants, but not transgenic lines. Our results indicate that transgenic *atzA* rice plants show tolerance to atrazine, and may be used as parental lines in future hybrid seed production.

## Introduction

Rice (*Oryza sativa*) is one of the most important staple food crops globally. According to the National Grain and Oil Information Center, the area of China planted with rice in 2012 was 3.0×10^7^ hm^2^, including 9.0×10^6^ hm^2^ of *japonica* rice [1]. *Japonica* rice is mainly planted in the northern region of the Qinling Mountains–Huai River, and its planted area has increased in recent years because of its high quality and good taste. *Japonica* rice production is currently dominated by conventional varieties, with hybrid rice accounting for only 3% of the cultivated area. On the other hand, *indica* hybrid rice represents 70–80% of the total planted area of *indica* rice [2]. Therefore, there is considerable potential for the development of *japonica* hybrid rice. An increase in the annual planted area of *japonica* hybrid rice from 3% to 50%, i.e., to reach 4.0×10^6^ hm^2^, is estimated to lead to the production of 3.5×10^9^ kg of high-quality grain (www.cngrain.com/Publish/qita/200503/207290), thereby contributing considerably to meet consumer's demand for high-quality food both in China and globally.

The three-line system is a traditional and effective production method for hybrid *japonica* rice seed [3]. The most widely used male sterile line in the system is BT-type cytoplasmic male sterile (CMS). However, the panicle of this line is loosely enclosed when heading, and this appearance closely resembles that of the maintainer. This makes it difficult for farmers to distinguish the BT-CMS line when eliminating off-type plants [2]. Furthermore, the BT CMS line has good restorability, and may therefore be easily pollinated with exotic pollens that contaminated during mechanical harvesting and storage of seeds, and also with exotic pollens from other plants [4]. The use of contaminated CMS lines in seed production results in decreased hybrid seed purity. Therefore, off-type contamination must be eliminated as early as possible. This is largely a manual process and requires considerable labor input, particularly in Asia. On the one hand, the need for increased labor will increase the price of hybrid seeds, while on the other hand, the increase in manual procedures may lead to the production of false hybrids. Furthermore, as the Chinese economy develops, increasing numbers of young men are leaving their home towns to seek work in the cities, leaving the elderly and women to work on the farms. The transformation of heavy and complex farming to light and simple farming is therefore becoming increasingly important. Thus, ensuring hybrid seed purity and reducing labor costs are two key issues in hybrid *japonica* rice seed production.

Genetic engineering, especially herbicide resistance engineering, provides an efficient means of controlling purity in hybrid seed production. Yan first proposed a strategy of utilizing herbicide resistance genes to chemically control purity in hybrid seed production [5]. Since then, two-line hybrid rice production has been extensively investigated [6]–[12] and progress has recently been reviewed [13]. Additionally, some transgenic hybrid rice combinations have been used in field trials [7], [8], [10], [12]. However, the research has mainly focused on the *bar* gene isolated from *Streptomyces hygroscopicus* [6], [8], [10], [12]; other genes, such as the EPSPS (5-enolpyruvylshikimate-3-phosphate synthase) gene from *Agrobacterium* strain CP4, and the protoporphyrinogen oxidase gene from *Bacillus subtilis*, have rarely been investigated [11]. If the strategy is proven to be effective, chemical control of hybrid seed purity will be mainly dependent on an herbicide with a single mode of action, and this will hinder sustainable weed management.

Atrazine (6-chloro-N^2^-ethyl-N^4^-isopropyl-1,3,5-triazine-2,4-diamine) is a triazine herbicide, and is commonly used in maize, sorghum, and sugarcane fields [14]. By inhibiting electron transport to plastoquinone in the photosystem PSII, atrazine terminates photosynthesis and kills weeds [15]. Atrazine was once the most widely used herbicide worldwide, because of its low cost and high effectiveness [14]. We previously isolated an atrazine chlorohydrolase gene (*atzA*) from a soil bacterium *Pseudomonas* ADP [16], and modified this gene by using directed evolution, to improve the enzymatic activity [17]. In the present study, we transferred the improved *atzA* gene into breeding hybrid *japonica* parental lines. Our results indicate that the transgenic *atzA* rice lines show tolerance to atrazine, and may be used as parental lines to chemically improve seed purity in hybrid seed production.

## Materials and Methods

### Construction of plant expression vector

Ubiquitin promoter and an improved atrazine chlorohydrolase gene *atzA*-22-4 [17] were respectively amplified from pSTARLING, an RNAi intermediate vector for monocots (a kind gift from the Commonwealth Scientific and Industrial Research Organization, Australia), and *AtzA*-22-4 using primers SL-Ubi-F/SL-Ubi-R and atzA-TJ-F/atzA-TJ-R (Table 1). After respectively inserted into TaKaRa pMD19T-simple vector and confirmed by sequencing, the pAtzA-22-4-19T-simple was restricted with *Eco*RV to collect the 1.4 kb fragment, and then lignated with pUbi-19T-simple. The resulting plasmid was restricted with *Sac*I/*Spe*I to collect the 3.4 kb fragment, and then ligated with pCAMBIA1301. The recombinant plasmid p1301-ubi-22-14 was then introduced into *Agrobacterium tumefaciens* EHA105 by the freeze-thaw method [18].

**Table 1:** Primers used in this study.

Primer	Sequence (5′→3′)
SL-Ubi-F	tgagctcctgcagtgcagcgtgacccggtcgt, with *Sac*I site
SL-Ubi-R	tgatatcctgcagaagtaacaccaaacaacag, with *Eco*RV site
atzA-TJ-F	tgatatcatgcaaacgctcagcatccag, with *Eco*RV site
atzA-TJ-R	tgatatcactagtctagaggctgcgccaagctg, with *Eco*RV and *Spe*I site
SL-Ubi-C-F	cccgccgtaataaatagacac
SL-Ubi-C-R	accacaccacatcatcacaac

### Genetic transformation and plant regeneration

*Oryza sativa* L. Nipponbare and *japonica* hybrid rice parental lines in the three-line system, Jindao7 (maintainer), Jindao8 (maintainer) and Jinhui3 (restorer) were used for transformation. Mature seeds were dehulled, surface-sterilized and placed on NB medium (N6 macro elements, B5 micro elements and vitamins) supplemented with 2 g/L proline, 3 mg/L 2, 4-D and 300 mg/L casein hydrolysate in dark at 28°C. After 2–3 weeks, the scutellum-derived calli were excised and subcultured every four weeks on the same medium but with 0.5 g/L proline, 2 mg/L 2, 4-D in dark at 28°C. The highly embryogenic compact calli (3–5 mm in diameter) that subcultured for less than five generations, were selected and co-cultivated with *A*. *tumefaciens* EHA105 harboring p1301C-ubi-22-14 on the co-cultivation medium (subculture medium but with 100 µM acetosyringone) for 3 days in dark at 28°C. Following that, the explants were then transferred into selection medium (subculture medium but with 50 mg/L hygromycin and 500 mg/L cefotaxime) in dark at 28°C for selection. After two cycles of selection, hygromycin-resistant calli were transferred onto pre-regeneration medium (NB medium with 0.5 g/L proline, 2 mg/L 6-BA, 1 mg/L NAA, 5 mg/L ABA, 300 mg/L casein hydrolysate and 50 mg/L hygromycin) for 14 to 21 days in dark at 28°C, then to regeneration medium (pre-regeneration medium but without 5 mg/L ABA) for 30 days under 54 µmol/m^2^/s light at 28°C and finally to the rooting medium (MS medium with 1 mg/L IBA) for 15 days under light at 28°C.

### Molecular analysis of transgenic lines

Genomic DNA was isolated from young leaves using a modified CTAB method [19]. PCR was performed to preliminarily select the transformed plants using primers atzA7/atzA8 [20] and SL-Ubi-C-F/SL-Ubi-C-R (Table 1). RT-PCR was performed to further confirm the expression of *atzA* in the PCR-positive transformed plants. Total RNA was extracted from young leaves using an RNAultra Extraction Kit (Qiagen). cDNA was synthesized using atzA8 and oligo(dT) as primers and SuperScript™^ II RNase H− (Invitrogen) as reverse transcriptase. RT-PCR was amplified using the cDNA as template and atzA7/atzA2 [20] as primers. *β*-actin (AB047313) was also amplified as the internal control using actin-R/actin-F as primers [21].^

### Germination and seedling growth in the presence of atrazine

Fifty seeds from each transgenic line and the respective control plant were directly sown on the surface of filter paper in plates containing 0, 75 or 150 µM atrazine. Seeds were placed in a growth chamber at 28°C with 16 h of 54 µmol/m^2^/s light per day. Germination (based on radicles >2 mm) was recorded daily and the cumulative values at day 3 and day 7 were calculated to represent as germination potential and germination percentage. The shoot length, root length and their biomass were measured after 7 days. For seedling growth test, seven-day old seedlings germinated in absence of atrazine were placed on filter paper in pots, and incubated in Kimura B nutrition solution [22] containing 0, 75 or 150 µM atrazine in growth chamber described above. During the period, the nutrition solution containing the respective concentration of atrazine was added to keep the filter paper wet. The growth parameters as described above were measured after 10 days.

### Soil-grown transgenic T_2_ lines in the presence of atrazine

Two-week old seedlings of similar size that germinated in absence of atrazine were transplanted into pots containing 1.12 kg of soil with 0, 2, or 5 mg/kg of atrazine. Nine plants were planted in each pot, and irrigated with the same amount of water every day and with Kimura B nutrition solution [22] twice a week. Plants were incubated in the greenhouse in a 14-h light/10-h dark cycle (28/25°C) at 300 µmol m^−2^s^−1^ light and 75% relative humidity. Plant growth and chlorophyll content were measured at 15 -day intervals. For chlorophyll content analysis, approximately 10 mg of leaves was extracted with 5 ml acetone and then quantified the absorbance at 663.6 nm and 646.6 nm, as described by Porra et al. [23]. Chlorophyll a = 12.25×*A*_663.6_–2.55×*A*_646.6_, Chlorophyll b = 20.31×*A*_646.6_–4.91×*A*_663.6_.

### HPLC analysis of atrazine and its metabolite hydroxyatrazine in the plant and soil

Atrazine and hydroxyatrazine in the rice leaves and soil were also determined in the soil-grown experiment. One gram of leaves, or five grams of soil samples that collected from the soil layer mixture as thick as possible, were extracted with 5 mL dichloromethane. After 30 min of incubation at room temperature, the mixture was centrifuged at 15,294 *g* for 10 min. The supernatant was transferred to a new tube and filtered through a 0.2-µm filter and then subjected to CoM 6000 HPLC system analysis on an analytical C18 column (5 µm, 250 mm×4.6 mm) at 30°C with linear gradients phase as follows: 0 to 6 min, 10% to 25% acetonitrile; 6 min to 21 min, 25% to 65% acetonitrile; 21 min to 23 min, 65% to 100% acetonitrile; and 23 min to 25 min, 100% acetonitrile [24] at the wavelength of 228 nm.

### Spraying atrazine to transgenic T2 lines

Two-week old seedlings from the germination experiment were also transplanted into pots with soil. Each pot contained 16 plants. After 30 days' growth, the last second leaves were taken and cut into 2–3 cm section, soaked in 0, 75 and 150 µM atrazine, and incubated at 25°C under light for 2 days. The color of the leaves was observed every day. After 40 days' growth, each pot of plants was sprayed with 20 ml of 0.15% atrazine solution.

### Statistical analysis

All the experiments were performed for three times. Significant difference between the specific transgenic line and the respective control was performed using independent-samples *t*-test at 95% or 99% confidence with IBM SPSS Statistics 11.0. Values indicated by * or ** represented significantly difference at *P*<0.05 or *P*<0.01.

## Results

### Selection of transgenic *atzA* plants

PCR analysis revealed that 18 (100%) Nipponbare transformants, 15 out of 18 (83%) Jindao7 transformants, 14 out of 16 (88%) Jinhui1 transformants, and 17 (100%) Jindao8 transformants were positive. RT-PCR analysis further confirmed the expression of *atzA* in the PCR-positive lines (data not shown). Subsequently, the self-pollinated seeds (T_1_ progenies) from each plant were germinated in the presence of hygromycin. Transgenic lines that showed a segregation pattern of 3∶1 resistant/sensitive in the germination test were selected to produce the T_2_ generations. Individual plants, whose herbicide tolerance did not segregate in the germination test, were considered as homogenous lines and selected. For simplicity, in further research, we used only two or three independent transgenic T_2_ lines for each genotype.

### Germination of transgenic *atzA* lines in the presence of atrazine

**Figure 1:**
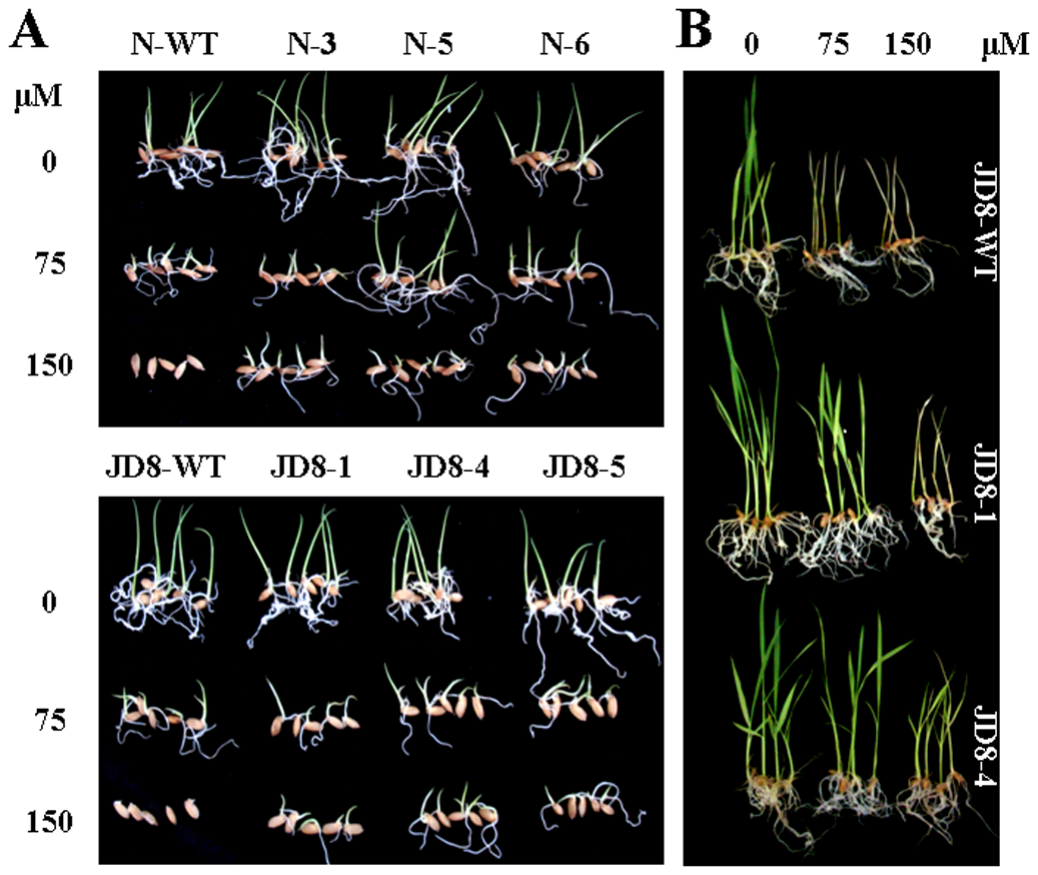
Germination and seedling growth of transgenic *atzA* rice lines in the presence of atrazine. (**A**) Representative images of seeds from the transgenic lines and the respective controls sown on plates containing 0, 75 or 150 µM atrazine for 7 days. (**B**) Representative images from seven-day old seedlings germinated in absence of atrazine were transplanted into pots containing 0, 75 or 150 µM atrazine for 10 days. For simplicity, only images of transgenic Jindao8 (JD8) and/or Nipponbare (N) were shown here.

To investigate the atrazine tolerance of transgenic lines during germination, we germinated seeds of three lines of each genotype and the respective controls in the presence of atrazine (Fig. 1A, Table 2). The germination potential of the wild types decreased as the atrazine concentration increased (58.3–98%, 22.4–64.2%, and 0%, respectively, in 0 µM, 75 µM, and 150 µM atrazine); on the other hand, the germination percentage significantly decreased only in presence of 150 µM atrazine (95.2–100%, 72.1–96.7%, and 0–42.2%, respectively, in 0 µM, 75 µM, and 150 µM atrazine). In the presence of the investigated atrazine concentrations, the highest germination potential was determined for Nipponbare, followed by Jinhui1, Jindao8, and Jindao7. In the presence of 0 µM or 75 µM atrazine, the germination potentials and percentages of almost all of the transgenic lines did not differ significantly from those of the respective controls. The exceptions were the germination potential for Jindao8 in the presence of 75 µM atrazine and the germination percentage for Jinhui1 in the presence of 75 µM atrazine. On the other hand, in the presence of 150 µM atrazine, all of the transgenic lines showed significantly higher germination potentials and germination percentages than did the respective controls. Moreover, two of the wild types (Nipponbare and Jinhui1) failed to germinate or only rarely germinated at day 3 or day 7. Interestingly, in the presence of 150 µM atrazine, the highest germination percentage was determined for Jindao7, followed by Jindao8, Jinhui1, and Nipponbare; on the other hand, Jindao7 showed the lowest germination potential. When germinated in the presence of atrazine, all of the transgenic lines produced larger seedlings (with taller shoots and longer roots) than did the respective control plants (Fig. 1A). However, the presence of atrazine significantly inhibited the growth of transgenic lines and wild types (Fig. 1A).

**Table 2:** Germination of transgenic *atzA* rice lines in the presence of atrazine.

Line	0 µM		75 µM		150 µM	
	3 d	7 d	3 d	7 d	3 d	7 d
N^a^-WT	98.0±2.0	100±0.0	54.0±1.6	94.5±1.4	0.0±0.0	0.0±0.0
N-3	98.0±1.6	94.2±0.0	60.0±2.2	98.2±1.1	64.3±1.6**	96.2±1.8**
N-5	96.0±2.0	100±0.0	64.0±1.3	92.1±1.5	62.3±2.6**	92.6±1.5**
JD7-WT	58.3±1.6	100±0.0	24.3±2.1	90.2±2.3	0.0±0.0	42.2±1.1
JD7-2	56.0±1.1	98.1±1.4	22.3±1.7	88.2±2.1	2.3±0.1**	88.5±1.6**
JD7-4	62.3±2.2	98.6±1.6	26.3±1.3	90.2±0.8	2.4±0.2**	86.5±1.3**
JH3-WT	86.3±2.0	95.2±2.8	64.2±2.2	72.1±1.1	0.0±0.0	4.5±0.0
JH-3	82.3±1.2	94.2±1.8	58.7±1.6	90.2±1.2*	32.3±0.9**	78.6±1.2**
JH3-5	88.3±3.2	92.2±1.3	66.3±2.7	94.1±1.0*	38.7±1.1**	80.5±1.2**
JD8-WT	82.0±4.2	98.7±0.8	22.4±1.6	96.7±1.0	0.0±0.0	26.5±1.3
JD8-1	78.3±2.5	99.2±0.8	48.3±1.1**	97.2±0.2	34.3±0.7**	94.5±2.8**
JD8-4	78.0±2.6	100±0.0	54.0±2.6**	98.3±1.0	35.2±1.6**	96.8±1.3**

Note: In each independent treatment 50 seeds were used for the experiment. Values are shown as mean ±SEM (*n* = 3). * or ** indicates significant difference (*P*<0.05) or highly significant difference (*P*<0.01) between the treatment and the control.

a N, JD7, JH3 and JD8 were the abbreviations of Nipponbare, Jindao7, Jindao8 and Jinhui3, respectively. For simplicity, only two transgenic lines for each genotype were shown here.

### Seedling growth of transgenic *atzA* lines in the presence of atrazine

To investigate the tolerance of transgenic lines to atrazine, we transplanted 7-day old seedlings germinated in the absence of atrazine, to pots of soil containing different concentration of atrazine (Fig. 1B and Table 3). In the absence of atrazine, we determined no differences in shoot or root growth between any of the transgenic lines and the respective controls. However, in the presence of 75 µM atrazine, all of the transgenic lines (except for Jindao8) were significantly taller than were the respective controls; further, all of the transgenic lines (except for Jinhui1) produced significantly more root biomass than did the respective controls. On the other hand, in the presence of 150 µM atrazine, shoot and root growth of almost all of the transgenic lines and the respective controls were significantly inhibited. The exceptions were the shoot growth of Nipponbare and the root growth of Jinhui1–5. Plants became yellowish, and subsequently rotted and died after 10 days (Fig. 1B).

**Table 3:** Seedling growth performance of transgenic *atzA* rice lines in the presence of atrazine.

Line	0 µM		75 µM		150 µM	
	Shoot length (cm)	Root biomass (mg)	Shoot length (cm)	Root biomass (mg)	Shoot length (cm)	Root biomass (mg)
N a-WT	9.90±1.37	32.00±3.16	6.54±0.86 (66.0)^a^	21.00±2.55 (65.6)	5.07±1.17 (51.2)	21.75±3.77 (68.0)
N-3	10.55±1.49	30.25±3.50	9.56±2.08* (90.7)	29.50±3.14** (97.5)	7.55±1.20* (71.6)	21.50±6.40 (71.1)
N-5	8.78±2.42	32.75±4.50	8.94±1.33* (101.8)	29.85±1.83** (91.2)	9.39±0.51** (106.9)	21.00±2.71 (64.1)
JD7-WT	8.47±0.37	28.50±2.56	5.28±0.57 (62.4)	20.00±3.10 (70.2)	6.54±0.61 (77.2)	17.75±5.19 (62.3)
JD7-2	9.40±2.61	31.50±3.00	7.08±0.78* (75.3)	32.50±2.65** (103.2)	5.81±0.56 (61.8)	17.00±3.46 (54.0)
JD7-4	7.99±1.65	27.50±3.35	8.16±1.27* (102.2)	27.50±2.65** (100.0)	6.78±0.53 (84.8)	17.75±4.19 (64.5)
JH3-WT	9.83±1.25	24.00±3.07	5.80±0.43 (59.0)	21.75±3.36 (90.6)	4.62±0.42 (47.0)	13.25±6.24 (55.2)
JH-3	10.44±1.47	26.75±2.75	8.27±0.59* (79.2)	24.25±2.22* (90.7)	5.12±0.61 (49.0)	15.25±2.63 (57.0)
JH3-5	8.89±1.58	25.50±3.57	8.25±0.78* (92.8)	23.25±2.87* (83.8)	6.04±0.19* (67.9)	22.00±3.37** (86.3)
JD8-WT	7.88±0.93	26.75±3.55	7.20±1.85 (91.4)	21.75±2.87 (81.3)	6.43±0.35 (81.5)	12.25±3.46 (45.8)
JD8-1	8.11±1.51	27.50±2.15	7.18±0.40 (88.6)	27.50±1.52** (100.0)	5.65±1.12 (69.6)	11.50±1.91 (41.8)
JD8-4	7.90±0.72	27.75±2.53	7.64±0.69 (96.7)	27.75±1.79** (100.0)	5.64±0.78 (71.3)	11.75±2.99 (42.3)

Note: The parameters were recorded after 10 days. Values are shown as mean ± SEM (*n* = 3). * or ** indicates significant difference (*P*<0.05) or highly significant difference (*P*<0.01) between the treatment and the control.

a N, JD7, JH3 and JD8 were the abbreviations of Nipponbare, Jindao7, Jindao8 and Jinhui3, respectively.

b Data in the bracket are the percentage of the treated values to the mock values.

### Tolerance of soil-grown transgenic *atzA* lines to atrazine

To further investigate the tolerance of transgenic lines to atrazine, we transplanted 15-day-old seedlings germinated in the absence of atrazine, to pots of soil containing 0 mg/kg, 2 mg/kg, or 5 mg/kg of atrazine. We measured the plant height (as a non-destructive assay of plant growth) and chlorophyll content at 15-day intervals.

**Figure 2:**
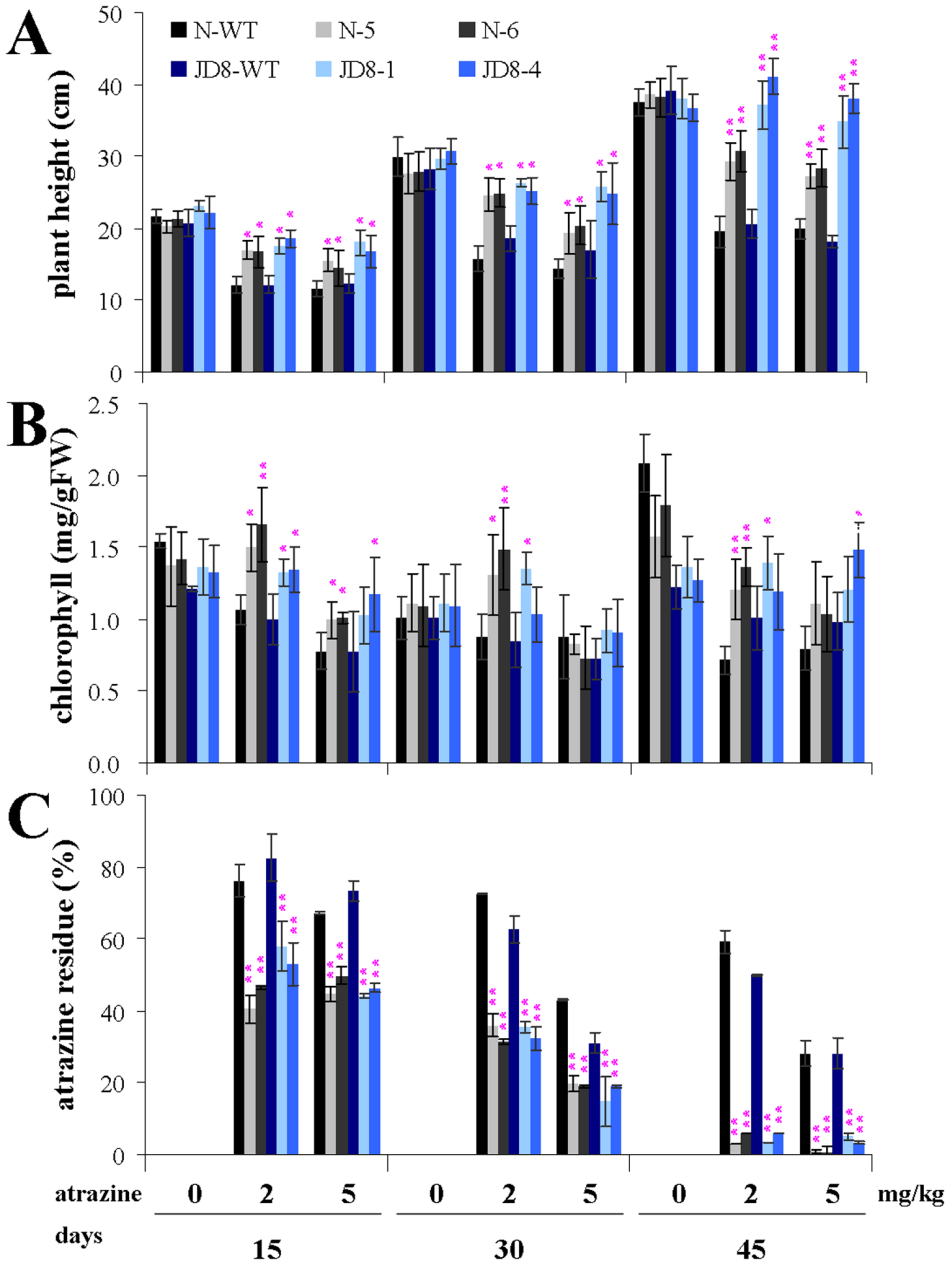
Growth and physiology of transgenic *atzA* rice lines in the presence of atrazine. Fifteen-day old seedlings germinated in absence of atrazine were transplanted into pots with 1.12 kg soil containing 0, 2 or 5 mg/kg of atrazine. (**A**) plant height; (**B**) chlorophyll content; and (**C**) atrazine residue were measured at 15-day intervals. Values are shown as mean ±SEM (*n* = 3). * or ** over the bar indicates significant difference (*P*<0.05) or highly significant difference (*P*<0.01) between the treatment and the control. For simplicity, only data of transgenic Nipponbare (N) and Jindao8 (JD8) were shown here.

In the absence of atrazine, we determined no significant differences in plant height between the transgenic lines and the respective controls (Fig. 2A). In soil containing 2 mg/kg or 5 mg/kg of atrazine, all the transgenic lines were significantly taller than were the respective control plants. However, the presence of atrazine significantly inhibited the growth of transgenic lines and control plants (by 68.1–111.9% and 46.4–68.2%, respectively). In contrast to the control plants, all of the transgenic lines (except for the Jindao8) were slightly taller in soil containing 2 mg/kg of atrazine than in soil containing 5 mg/kg of atrazine. The growth of transgenic lines gradually recovered with an increase in the growth time. Further, three of the strongest transgenic lines (Jindao7–4, Jinhui1–5, and Jindao8–4) completely recovered after 45 days of growth (data not shown).

Atrazine may disrupt the electron flow of photosystem II and destroy photosynthetic pigments in plant leaves [15]. In the absence of atrazine, we observed no difference in chlorophyll content between transgenic lines and control plants (Fig. 2B). However, in soil containing 2 mg/kg or 5 mg/kg of atrazine, the transgenic lines retained more chlorophyll than did the respective control plants. With a few exceptions, the chlorophyll content of the transgenic lines decreased significantly in soil containing 5 mg/kg of atrazine, but not in soil containing 2 mg/kg of atrazine; on the other hand, the chlorophyll content of the control plants decreased significantly under both conditions. For each genotype grown in soil containing the same atrazine concentration, we observed no difference in chlorophyll content at different growth stages. After 45 days, the chlorophyll contents of the three strongest lines (Jindao7–4, Jinhui1–5, and Jindao8–4) were almost identical when grown in soil containing 5 mg/kg of atrazine and when grown in the absence of atrazine (data not shown).

### Ability of soil-grown T_2_ transgenic lines to degrade atrazine

To investigate the ability of the soil-grown transgenic plants to degrade atrazine, we determined the atrazine residue in the soil at 15-day intervals, after growth of seedlings in pots of soil containing 0 mg/kg, 2 mg/kg, or 5 mg/kg of atrazine. We observed that the atrazine residue in the soil decreased with an increase in growth time, for transgenic and control plants (Fig. 2C). However, the decrease was greater for transgenic lines than for control plants. After treatment with 2 mg/kg of atrazine, the atrazine residue remaining in the soil was 2.9–7.0% for transgenic lines, and 44.0–59.2% for control plants; after treatment with 5 mg/kg of atrazine, the atrazine residue remaining in the soil was 0.8–8.7% for transgenic lines and 28.1–30.8% for control plant. For each genotype at the same growth stage, the atrazine residue in the soil was higher after growth in 2 mg/kg atrazine than after growth in 5 mg/kg atrazine. We also observed that hydroxyatrazine, not atrazine (only trace) appeared in the leaves of the transgenic plants (Fig. S1), indicating that atrazine was metabolized rather than accumulated in the transgenic plants.

### Utilization of transgenic plants in hybrid seed production

**Figure 3:**
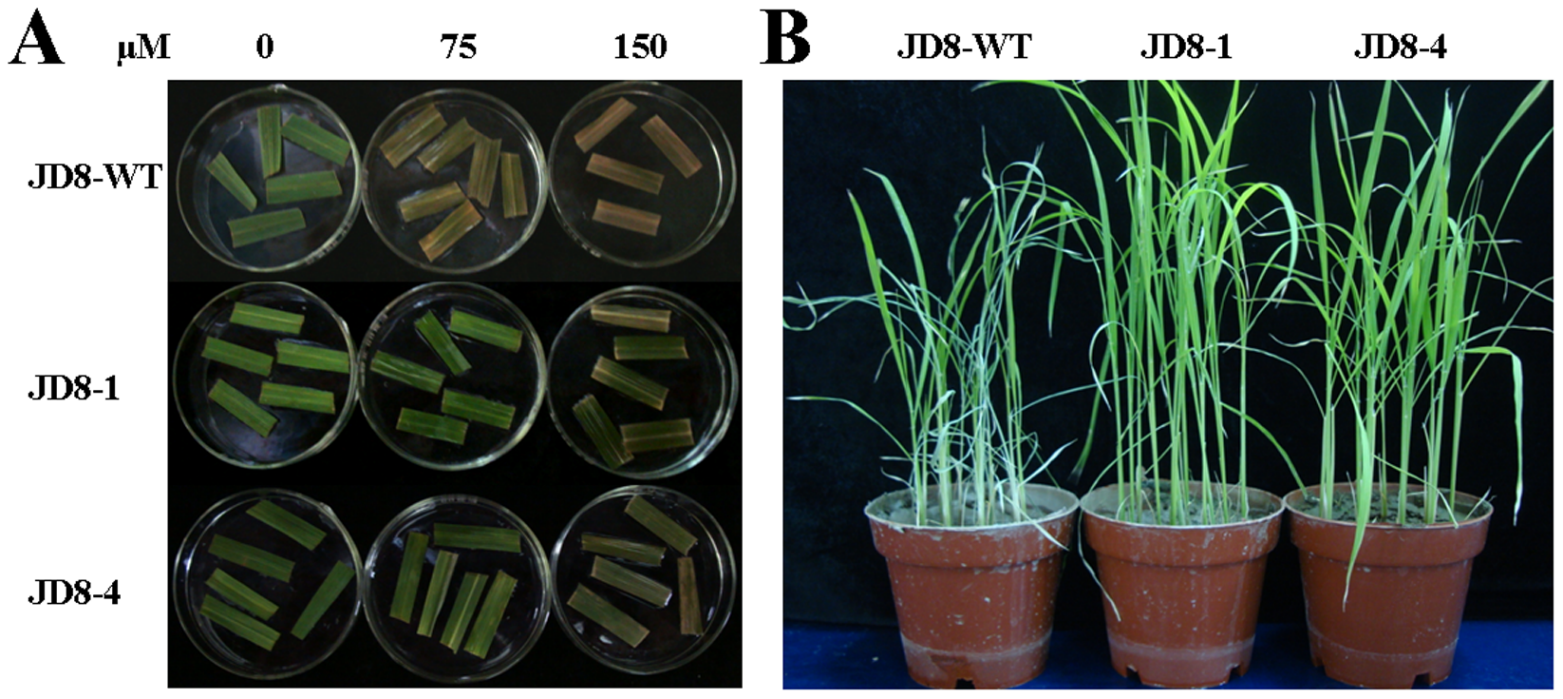
Tolerance of mature transgenic *atzA* plants to atrazine. (**A**) Representative images of leave sections in the presence of atrazine. Fifteen-day old seedlings were transplanted into pots containing soil. After 30 days' growth, the last second leaves were cut into 2–3 cm section, and soaked in 0, 75 and 150 µM atrazine, and incubated at 25°C under light for 2 days. (**B**) Representative images of mature plants after sprayed with atrazine. Fifteen-day old seedlings were transplanted into pots containing soil. After 40 days' growth, each pot of transgenic lines (9 plants) was sprayed with 20 mL of 0.15% atrazine solution. For simplicity, only data of transgenic Jindao8 (JD8) was shown here.

To investigate the tolerance of transgenic plants to the atrazine concentration used in weed control, we first examined the tolerance of leaf sections to atrazine solution. In the presence of 75 µM atrazine, the leaf sections of transgenic lines remained green, whereas those of control plants became bleached (Fig. 3A). In the presence of 150 µM atrazine, the leaf sections of transgenic lines became slightly bleached, but remained greener than those of control plants. We subsequently sprayed plants with 0.15% atrazine, and observed that wild-type plants became curled and withered after 6 days, whereas transgenic lines continued to grow well (Fig. 3B). Our results indicate that the transgenic rice plants showed tolerance to the atrazine concentration used in weed control.

## Discussion

Current research on the utilization of herbicide resistance genes in hybrid rice is focused on the two-line hybrid production system. Sterile lines are not stable in the two-line system, and therefore most *japonica* hybrid combinations are still produced by using three-line system. In the present study, we have developed atrazine-tolerant transgenic *japonica* rice parental lines with the potential to be used in future hybrid seed production.

Herbicide tolerance during germination is very important when utilizing transgenic *atzA* lines in hybrid seed production, because off-type plants should be eliminated as early as possible. Atrazine is a slightly water-soluble herbicide, with a saturated concentration of 153 µM. Kawahigashi [25] previously showed that an atrazine concentration of 100 µM did not affect the germination of rice plants. Therefore, we used two atrazine concentrations (75 µM and 150 µM) in the germination test, to evaluate the tolerance of transgenic rice lines. In contrast to Kawahigashi [25], we observed that the transgenic lines germinated well in the presence of atrazine whereas the respective controls did not. However, as the seedling growth of transgenic lines were also inhibited by atrazine, chemical control of contamination during germination may not be appropriate.

To evaluate the tolerance of seedlings to atrazine, we used 7-day-old seedlings germinated in the absence of atrazine as a starting material, to avoid cumulative effects derived from germination. We observed that almost all of the transgenic lines grew well in the presence of 75 µM atrazine with no difference from that in absence of atrazine. Therefore, 75 µM atrazine may be used for effective control of off-type plants during the early seedling growth stage. Our data also suggested spraying plants with 0.15% atrazine (equivalent to the standard dosage used in the field for weed control, i.e., 200–250 g 40% suspension concentrate in 30∼50 kg of water) at the subsequently vegetative growth stage could serve as an alternative means of chemically controlling off-type plants. However, we did not test the tolerance of transgenic *atzA* lines at the reproductive growth stage, which is important for the utilization in mechanical harvesting of hybrid seed production. Further studies are required to investigate whether the lethal dosage for untransformed parental lines is tolerated at the reproductive stage.

Atrazine degrades relatively slowly, with an average half-life of 4–57 weeks [26]. Therefore, application of this herbicide will inevitably lead to accumulation in the field, which may affect current crop growth or damage sensitive succession crops [27]. The transgenic rice lines were taller, and had higher chlorophyll contents than did the respective control plants when growing in the soil of 2 mg/kg or 5 mg/kg atrazine. Three of the transgenic lines (Jindao7–4, Jinhui1–5, and Jindao8–4) completely recovered after 45 days of growth in the presence of atrazine, suggesting that an atrazine concentration up to 5 mg/kg does not affect sustained growth of transgenic lines. Our data also suggested that almost all of the atrazine was degraded after growth of the transgenic rice plants for 45 days. This was also verified by the appearance of hydroxyatrazine in the leaves of transgenic plants. Besides, atrazine metabolite hydroxyatrazine could be directly determined in transgenic plants. However, as the leave, stem and root of the plants all possibly metabolize atrazine [28], it would be more complicated if we want to quantitatively evaluate the atrazine metabolized by plants. That is why we chose to determine the atrazine residue in the soil after growth of the transgenic plants in this study.

Utilization of herbicide resistance genes in hybrid seed production may be achieved by introducing the genes into a maintainer or restorer line in the three-line system. When an herbicide-resistant maintainer is obtained, its corresponding CMS line, which possesses herbicide resistance, may be created by using a backcross. The resulting herbicide-resistant CMS breeder seeds may be used to cross with the original maintainer (herbicide sensitive), to multiply the herbicide-resistant foundation seeds. During this process, the selfed maintainer and off-type plants may easily be eliminated by herbicide spraying, thus ensuring the purity of CMS seeds. The herbicide-resistant CMS line may also be used in mechanical harvesting, by spraying herbicide to kill the pollen plants after pollination, and harvesting seeds from the surviving CMS plants. A similar result may be achieved when crossing the herbicide-resistant CMS line with the herbicide-sensitive restorer line. Alternatively, the herbicide-resistant restorer line may be crossed with the herbicide-sensitive sterile line during the hybrid seed production process, by spraying herbicide to kill the off-type plants. Further, the herbicide-resistant maintainer and restorer lines may be useful for eliminating off-type plants, and for weed management when growing hybrid rice.

Field trials with genetically modified rice are strictly controlled in China, and therefore we were unable to evaluate our herbicide-resistant hybrid rice combinations on a field scale. Further studies are required to verify the validity of utilizing atrazine-tolerant transgenic lines in the field, and to determine whether herbicide-resistant hybrid rice combinations retain their original superiority in terms of yield. Nevertheless, the development of atrazine-tolerant transgenic *japonica* rice parental lines represents a valuable tool for future application in hybrid seed production.

## Supporting Information

Figure S1 **HPLC analysis for determination of atrazine and its metabolite hydroxyatrazine in leaves of transgenic and WT rice plants after grown in 5 mg/kg atrazine soil for 45 days.** (**A**) Standard of hydroxyatrazine (I) and atrazine (II). (**B**) Wild type (WT). (**C–E**) Transgenic Nipponbare (N-6), Jindao7 (JD7-4) or Jindao8 (JD8-4), respectively.
(TIF)

## References

[1] Peng C, Zhang H (2013) Analysis of paddy and rice markets at home and abroad in the first quarter of 2013 and its prospect. Agric Outlook (4): 4–9 (in Chinese, with English abstract).

[2] Shi KB, Deng HS (2004) Chinese *japonica* hybrid rice technology innovation seminar held in Sanya. Hybrid Rice 19: 76 (in Chinese)

[3] Li S, Yang D, Zhu Y (2007) Characterization and use of male sterility in hybrid rice breeding. J Integ Plant Biol 49: 791–804.

[4] Tang SZ, Zhang HG, Zhu ZB, Liu C, Li P, et al (2010) Application of HL type male sterile cytoplasm in *japonica* hybrid rice breeding. Chin J Rice Sci 24: 116–124 (in Chinese, with English abstract)

[5] Yan W (2000) Crop heterosis and herbicide. US Patent 6066779.

[6] Hu G, Xiao H, Yu Y, Zhu Z, Si H, et al (2000) *Agrobacterium*-mediated transformation of the restorer lines of two-line hybrid rice with *bar* gene. Chin J Appl Environ Biol 6: 511–515 (in Chinese, with English abstract)

[7] Li Y, Xu Q, Duan F, Liu G, Yan W (2000) Breeding of herbicide-resistant hybrid rice combinations. Hybrid Rice 15(6): 9–11.

[8] Xue S, Zhang W, Gong X, Shen L, Huang D, et al (2001) Breeding of isotype restorer line with *bar* gene from Miyang 46 and its combinations. J Zhejiang Agric Sci (4): 181–183 (in Chinese)

[9] Ramesh S, Nagadhara D, Pasalu IC, Kumari AP, Sarma NP, et al (2004) Development of stem borer resistant transgenic parental lines involved in the production of hybrid rice. J Biotechnol 111: 131–141. 15219400 10.1016/j.jbiotec.2004.04.004

[10] Xiong X, Tang L, Deng X, Xiao G (2004) A preliminary report on the experiments of herbicide-resistant two-line hybrid rice Xiang 125S/Bar 68-1. Hybrid Rice 19(5): 41–43.

[11] Wu F, Wang S, Li S, Zhang K, Li P (2006) Research progress on herbicide resistant transgenic rice and its safety issues. Mol Plant Breed 4: 846–852.

[12] Xiao G, Yuan L, Sun S (2007) Strategy and utilization of a herbicide resistance gene in two-line hybrid rice. Mol Breed 20: 287–292.

[13] Xiao G (2009) Recent advances in development of herbicide resistance transgenic hybrid rice in China. Rice Sci 16: 235–239.

[14] Udiković-Kolić N, Scott C, Martin-Laurent F (2012) Evolution of atrazine-degrading capabilities in the environment. Appl Microbiol Biotechnol 96: 1175–1189.. 23076592 10.1007/s00253-012-4495-0

[15] Rutherford AW, Krieger-Liszkay A (2001) Herbicide-induced oxidative stress in photosystem II. Trends Biochem Sci 26: 648–653. 11701322 10.1016/s0968-0004(01)01953-3

[16] Cai B, Han Y, Liu B, Ren Y, Jiang S (2003) Isolation and characterization of an atrazine-degrading bacterium from industrial wastewater in China. Lett Appl Microbiol 36: 272–276. 12680937 10.1046/j.1472-765x.2003.01307.x

[17] Wang Y, Li X, Chen X, Chen D (2013) Directed evolution and characterization of atrazine chlorohydrolase variants with enhanced activity. Biochemistry (Moscow) 78: 1104–1111. 24237144 10.1134/S0006297913100040

[18] Höfgen R, Willmitzer L (1988) Storage of competent cells for *Agrobacterium* transformation. Nucleic Acids Res 16: 9877. 3186459 10.1093/nar/16.20.9877 PMC338805

[19] Loh JP, Kiew R, Kee A, Gan LH, Gan YY (1999) Amplified fragment length polymorphism (AFLP) provides molecular markers for the identification of *Caladium bicolor* cultivars. Ann Bot 84: 155–161.

[20] Wang H, Chen X. Xing X, Hao X, Chen D (2010) Transgenic tobacco plants expressing *atzA* exhibit resistance and strong ability to degrade atrazine. Plant Cell Rep 29: 1391–1399.. 20960204 10.1007/s00299-010-0924-7

[21] Chen D, Chen H, Zhang L, Shi X, Chen X (2014) Tocopherol-deficient rice plants display increased sensitivity to photooxidative stress. Planta 239: 1351–1362.. 24691571 10.1007/s00425-014-2064-8

[22] Ma J, Takahashi E (1990) Effect of silicon on the growth and phosphorus uptake of rice. Plant Soil 126: 115–119.

[23] Porra RJ, Thompson WA, Kriedemann PE (1989) Determination of accurate extinction coefficients and simultaneous equations for assaying chlorophylls *a* and *b* extracted with four different solvents: verification of the concentration of chlorophyll standards by atomic absorption spectroscopy. Biochim Biophys Acta - Bioenergetics 975: 384–394.

[24] de Souza ML, Sadowsky MJ, Wackett LP (1996) Atrazine chlorohydrolase from *Pseudomonas* sp. strain ADP: gene sequence, enzyme purification, and protein characterization. J Bacteriol 178: 4894–4900. 8759853 10.1128/jb.178.16.4894-4900.1996 PMC178272

[25] Kawahigashi H, Hirose S, Ohkawa H, Ohkawa Y (2007) Herbicide resistance of transgenic rice plants expressing human CYP1A1. Biotechnol Adv 25: 75–84. 17156966 10.1016/j.biotechadv.2006.10.002

[26] Erickson LE, Lee KH, Sumner DD (1989) Degradation of atrazine and related *s*-triazines. Crit Rev Environ Control 19: 1–14.

[27] Rhine ED, Fuhrmann JJ, Radosevich M (2003) Microbial community responses to atrazine exposure and nutrient availability: linking degradation capacity to community structure. Microbiol Ecol 46: 145–160. 10.1007/s00248-002-1048-6 14708741

[28] Wang L, Samac DA, Shapir N, Wackett LP, Vance CP, et al (2005) Biodegradation of atrazine in transgenic plants expressing a modified bacterial atrazine chlorohydrolase (*atzA*) gene. Plant Biotech J 3: 475–486. 10.1111/j.1467-7652.2005.00138.x 17173634

