# Supplementary figures and images for: Evaluation of the Agronomic Performance of Atrazine-Tolerant Transgenic japonica Rice Parental Lines for Utilization in Hybrid Seed Production
Source: PLoS One. 2014 Oct 2;9(10):e108569. doi: 10.1371/journal.pone.0108569 (PMC4183513; doi:10.1371/journal.pone.0108569)

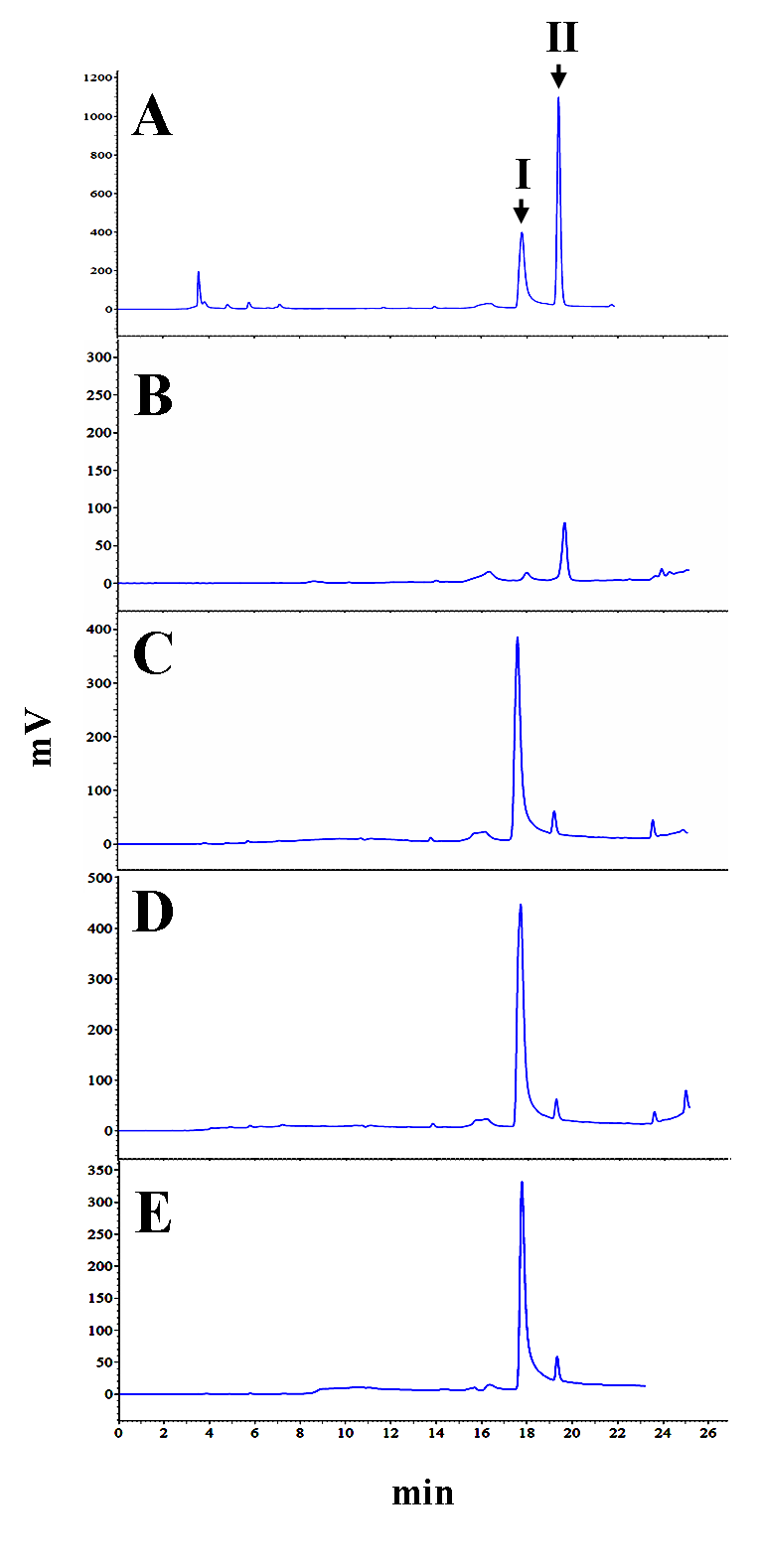

Supplement: Figure S1 — HPLC analysis for determination of atrazine and its metabolite hydroxyatrazine in leaves of transgenic and WT rice plants after grown in 5 mg/kg atrazine soil for 45 days. (A) Standard of hydroxyatrazine (I) and atrazine (II). (B) Wild type (WT). (C–E) Transgenic Nipponbare (N-6), Jindao7 (JD7-4) or Jindao8 (JD8-4), respectively. (TIF) [file pone.0108569.s001.tif]
